# Association between heaviness of cigarette smoking and serious psychological distress is stronger in women than in men: a nationally representative cross-sectional survey in Japan

**DOI:** 10.1186/s12954-021-00469-5

**Published:** 2021-03-04

**Authors:** Kimiko Tomioka, Midori Shima, Keigo Saeki

**Affiliations:** Nara Prefectural Health Research Center, Nara Medical University, 840 Shijo-cho, Kashihara, Nara 634-8521 Japan

**Keywords:** Psychological distress, Cigarette smoking, Tobacco, Smoking heaviness, Population-based study

## Abstract

**Background:**

Higher smoking prevalence in people with serious psychological distress (SPD) is well-recognized. However, gender and age differences in the association between heaviness of cigarette smoking and SPD have not been fully investigated.

**Methods:**

We used anonymized data from a nationally representative survey in Japan (33,925 men and 37,257 women). SPD was measured using the Kessler 6-item Psychological Distress Scale and defined as ≥ 13 points. Multiple logistic regression analyses stratified by gender and age-groups (20–44 years, 45–64 years, and ≥ 65 years) were used to estimate adjusted odds ratio (aOR) and 95% confidence interval (CI) for SPD.

**Results:**

After adjusting for sociodemographic confounders including education, equivalent household expenditures, and employment contract, women had a significant association between heavier smoking and more frequent SPD: compared to never-smokers, aORs (95% CIs) of ex-smokers, current light smokers who smoked 1–10 cigarettes per day (CPD), current moderate smokers 11–20 CPD, and current heavy smokers ≥ 21 CPD were 1.22 (0.92–1.63), 1.52 (1.25–1.84), 1.75 (1.46–2.09), and 2.22 (1.59–3.10), respectively (*P*-trend < 0.001). A significant positive association among women was consistent across all age-groups. Among men, there was no association between heaviness of cigarette smoking and SPD in all age-groups, and only current heavy smokers aged 20–44 years had a significantly higher OR for SPD (aOR, 1.37 [95% CI, 1.02–1.85]) than never-smokers.

**Conclusions:**

There was a positive association between heaviness of cigarette smoking and SPD only among women, but not among men. For female smokers experiencing mental disorders, there is a need not only to improve mental health services but also to improve smoking-cessation support.

## Background

Cigarette smoking is the most modifiable risk factor for premature deaths in the world [1]. Numerous studies, especially in Western countries, have reported that individuals experiencing psychological distress (i.e., common mental disorders such as symptoms of depressed and anxious mood or malaise), are more likely to have a higher prevalence of current cigarette smoking than the general population [2–8].

It is well known that current cigarette smoking prevalence is higher in men than in women and in younger age-groups than in older age-groups [1, 4, 6, 9], while the prevalence of serious psychological distress (SPD) is higher in women than in men [5, 6, 10, 11]. However, gender and age differences in the association between current cigarette smoking and SPD are not consistent with previous studies, which reported a stronger association for women than for men [3], no gender differences [4, 5], and a significant association only for women [12], young people [5], or older adults [4, 7]. The possible reasons for inconsistent results in previous studies may be as follows: (1) the differences in the age structure of people with SPD (e.g., SPD was prevalent in older age-groups [4] or in younger age-groups [3, 6], a U-shaped association with a nadir for persons aged 65–69 years [5], or an inverted U-shaped relationship with a peak for persons aged 45–64 years [8]); (2) an increasing trend in the number of people with SPD [7]; (3) a falling trend in cigarette smoking prevalence in the wake of the buildup of anti-smoking measures [1, 13]; and (4) the differences in the stages of the cigarette epidemic [14]. Moreover, to our knowledge, no studies have examined gender and age differences in an association between heaviness of cigarette smoking and SPD. Verifying gender and age differences in the association between heaviness of cigarette smoking and SPD can inform effective tobacco control efforts, which would specifically target high-risk groups, while also addressing the needs of the general population.

In the present study, using the latest anonymized data from a nationally representative cross-sectional survey in Japan, we investigated an association between heaviness of cigarette smoking (the number of daily cigarettes) and the prevalence of those with SPD. These relations were stratified by gender and age, controlling for potentially important socio-demographic cofounders, such as family size, housing tenure, marital status, education, equivalent household expenditures, employment contract, and chronic medical conditions.

## Methods

### Data source

The data used came from the 2013 Comprehensive Survey of Living Conditions (CSLC). The CSLC is conducted by the Ministry of Health, Labour and Welfare (MHLW) of Japan, and is an annual cross-sectional nationwide survey to collect data on national lifestyle, health, and welfare. A major survey is held once every three years. In other years, a minor survey which excludes smoking and SPD is implemented. In the 2013 CSLC, survey slips were distributed on June 6 to all households in 5,530 stratified random sampling districts (295,367 households). The response rate was 79.6%. On the basis of the Statistics Act, Article 36, the MHLW granted us a license to use the anonymized data from the 2013 CSLC for academic research. In March 2020, the 2013 data was the most of up-to-date anonymized dataset available to researchers. The anonymized data had personal information deleted and were resampled by the MHLW. Therefore, an anonymized dataset from the 2013 CSLC included 97,345 people in 38,882 households; the reduced size equivalent to that of minor surveys. For full details on the CSLC dataset, see the MHLW Web site [15] and previous studies [9, 16].

### Study participants

In Japan, cigarette smoking by persons aged < 20 years is prohibited by law. Therefore, the 2013 CSLC asked individuals of 20 years or more a question about smoking status. Additionally, people in a hospital/facility or who had received certification of long-term care need were exempt from answering questions about health status and lifestyles. Therefore, our analyses were limited to community-dwelling adults aged 20 and older without long-term care insurance certification; we excluded 26,163 persons from our analyses due to being aged < 20 years (*n* = 17,620), admission to a hospital/nursing home or certification of long-term care need (*n* = 3,083), missing data on age and/or admission (*n* = 1,360), and missing data on smoking status and/or SPD (*n* = 4,100). The final number of participants included in this study, then, was 71,182 persons aged 20 years and older (33,925 men and 37,257 women).

### Measures

#### Heaviness of cigarette smoking

For smoking status, participants were asked about the presence or absence of current cigarette smoking and their smoking history. In addition, current smokers were asked the mean number of cigarettes smoked per day and were categorized into the following three groups: light (i.e., 1–10 cigarettes per day [CPD]), moderate (i.e., 11–20 CPD), and heavy (i.e., 21 or more CPD). To assess heaviness of cigarette smoking, smoking was classified into five groups: never-smokers, ex-smokers, current light smokers, current moderate smokers, and current heavy smokers.

#### Serious psychological distress (SPD)

SPD was surveyed using the Japanese version of the Kessler 6-item Psychological Distress Scale (K6) with demonstrated reliability and validity [17]. The K6 consists of six items about non-specific psychological distress and asks about negative emotional states during the past 30 days (score range 0–24); a higher score indicates a higher level of SPD [18]. Based on the cutoff point which has demonstrated adequate validity in detecting people with serious mental illness in the general population [19], participants were dichotomized into those with SPD (a score of 13 or more) and those without SPD (0–12 score). This cutoff point of 13 has been used in previous studies on SPD [3, 7].

#### Covariates

Previous studies indicate that low socioeconomic position, such as living alone, never or previously married, low educational level, and poor economic status, is associated with both higher prevalence of SPD and increased smoking prevalence [9, 20–24]. Recent research has reported that housing tenure is considered one of the important factors in determining mental health [25, 26]. Additionally, Inoue et al. examined the association of occupation, employment contract, and company size with mental health among Japanese employees, and found that only employment contract was associated with SPD, suggesting that employment contract may be an important indicator for Japanese social class [20]. Therefore, in this study, the following variables were included as covariates that may correlate with smoking status and SPD: age, family size, housing tenure, marital status, education, equivalent household expenditures (EHE), employment contract, and chronic medical conditions.

Family size (number of people in a family) was categorized into 1 (living alone), 2, 3–4, and ≥ 5. Housing tenure was dichotomized into owner-occupiers and renters. Marital status was categorized into married, never-married, and widowed/divorced. Education (years of schooling) was categorized into junior high school (≤ 9 years), high school (10–12 years), junior college (13–15 years), and college or higher (≥ 16 years). EHE (Japanese thousand yen per month) were divided into three categories by tertiles: low (≤ 105), middle (106–156), and high (≥ 157). Employment contracts were categorized into regular employees, part-time employees, temporary/contract employees, self-employed, and non-working. Chronic medical conditions were defined as persons with at least one disease under treatment for diabetes mellitus, hypertension, stroke, myocardial infarction, or cancer; these five diseases reportedly are associated with SPD among community-dwelling adults aged ≥ 40 years in Japan [5].

We checked that there were no problems of multicollinearity of all covariates; in this study, the variance inflation factor (VIF) showed a maximum score of 1.32 and none of the covariate variables had more than 5 of VIF. To deal with missing data on the covariates, we used a category entitled “missing” and considered the effect of no response to questions on the covariates. Further details about the covariates are shown in Additional file 1: Table 1 for men and Table 2 for women. Additionally, prevalence of SPD according to gender, age, and basic characteristics is shown in Additional file 1: Table 3.

### Statistical analysis

The difference in the prevalence of current cigarette smoking or SPD between men and women was analyzed using the chi-squared test. A trend test to detect the increased prevalence of current cigarette smoking or SPD was conducted using the Mantel-extension method.

The adjusted odds ratio (aOR) with 95% confidence interval (CI) for SPD was calculated using multivariate logistic regression models after simultaneously adjusting for all covariates, stratified by gender (men and women) and age categories (aged 20–44 years, aged 45–64 years, and aged ≥ 65 years). The dependent variable was SPD (a total K6 score of ≥ 13 points). Independent variables were heaviness of cigarette smoking (never-smokers, ex-smokers, current light smokers 1–10 CPD, current moderate smokers 11–20 CPD, and current heavy smokers ≥ 21 CPD).

We performed sensitivity analyses which excluded people who received medical treatment for a mental disorder including depression (*n* = 1,351) and people who had uncertain treatment status for a mental disorder (*n* = 266). We also examined the interaction effect between heaviness of smoking and socio-demographic variables, such as family size, housing tenure, marital status, education, EHE, employment contract, and chronic medical conditions.

The significance level was set at *P* < 0.05. All analyses were performed with the SPSS version 24.0 (IBM Corporation, Armonk, NY, USA).

## Results

In this study, the prevalence of current smokers was 34.2% in men and 10.8% in women, showing a significant gender difference (*P* < 0.001, Chi-squared test). The prevalence of SPD was 3.5% in men and 4.5% in women, showing a significant gender difference (*P* < 0.001, Chi-squared test).

Among both genders, the younger the participants were, the more frequently they had current cigarette smoking and SPD (*P* for trend < 0.001 in both smoking and SPD, Mantel-extension method). Older age-groups had a higher prevalence of home ownership, junior high school education, and chronic medical conditions, while younger age-groups had a higher prevalence of regular employment. People aged 45–64 had the lowest prevalence of low EHE (Table 1).

**Table 1:** Characteristics of analyzed participants by gender and age

	Men (*n* = 33,925)			Women (*n* = 37,257)		
	20–44 years (*n* = 12,851) *n* (%)	45–64 years (*n* = 12,126) *n* (%)	≥ 65 years (*n* = 8948) *n* (%)	20–44 years (*n* = 13,558) *n* (%)	45–64 years (*n* = 12,920) *n* (%)	≥ 65 years (*n* = 10,779) *n* (%)
Family size: one (i.e., living alone)	1691 (13.2)	1482 (12.2)	977 (10.9)	963 (7.1)	1038 (8.0)	2302 (21.4)
Housing tenure: owner-occupiers	7665 (59.6)	9392 (77.5)	7614 (85.1)	8339 (61.5)	10,264 (79.4)	8974 (83.3)
Marital status: married	6474 (50.4)	9559 (78.8)	7592 (84.8)	7573 (55.9)	10,138 (78.5)	6030 (55.9)
Education: junior high school^a^	636 (4.9)	1004 (8.3)	2523 (28.2)	436 (3.2)	909 (7.0)	3754 (34.8)
Equivalent household expenditures: low^b^	4593 (35.7)	3408 (28.1)	2911 (32.5)	4843 (35.7)	3552 (27.5)	3967 (36.8)
Employment contract: regular employees	8418 (65.5)	6353 (52.4)	363 (4.1)	4276 (31.5)	2256 (17.5)	135 (1.3)
Chronic medical conditions: present^c^	302 (2.4)	2682 (22.1)	4069 (45.5)	199 (1.5)	1993 (15.4)	4259 (39.5)
Smoking status: current smokers	5149 (40.1)	4691 (38.7)	1763 (19.7)	1976 (14.6)	1575 (12.2)	461 (4.3)
Serious psychological distress: present	654 (5.1)	356 (2.9)	194 (2.2)	846 (6.2)	510 (3.9)	319 (3.0)

^a^≤ 9 years of schooling

^b^Lower tertile (≤ 105 Japanese thousand yen per month)

^c^Defined as persons with at least one disease under treatment for diabetes mellitus, hypertension, stroke, myocardial infarction, or cancer

**Fig. 1:**
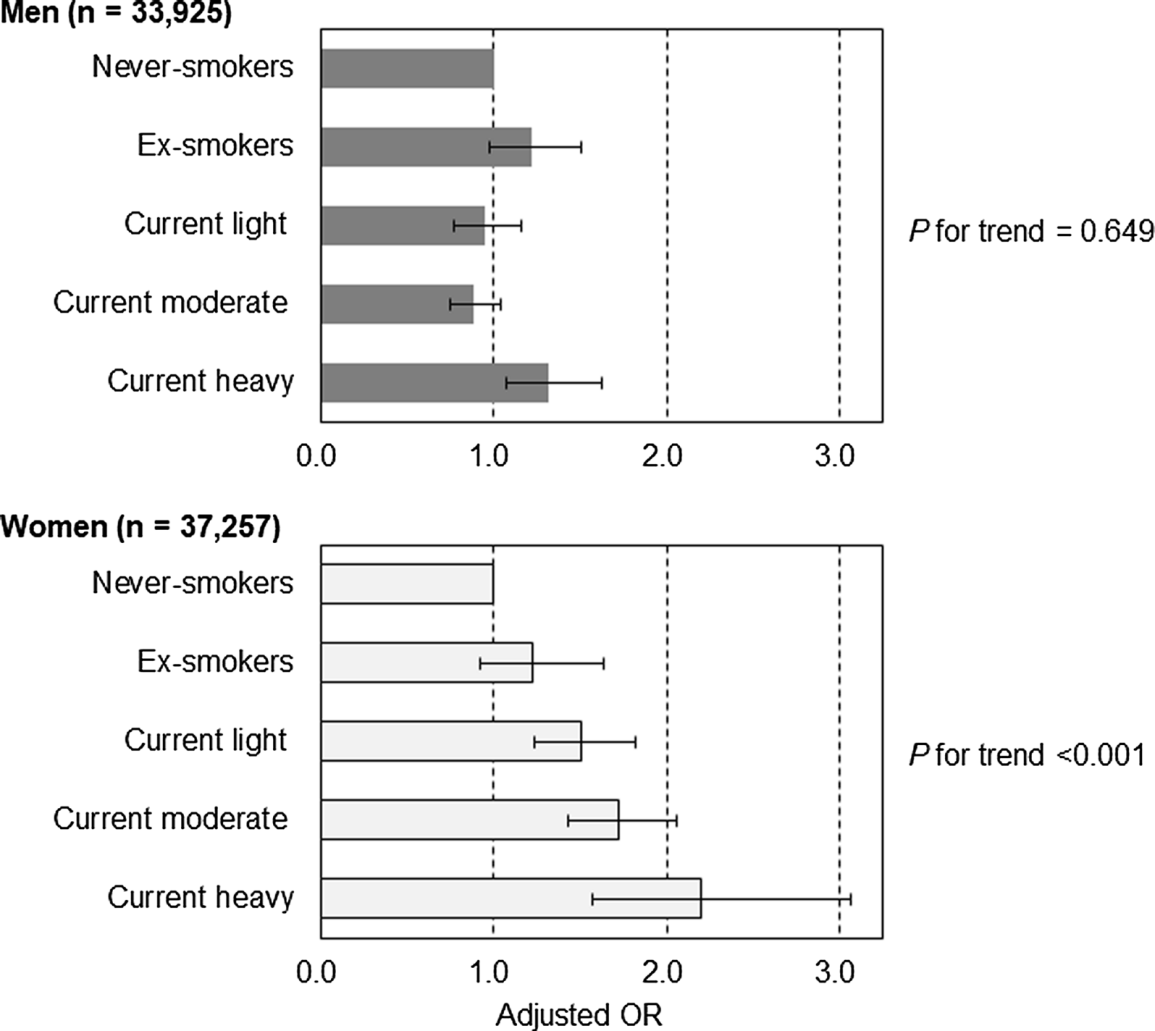
Adjusted odds ratio (OR) of heaviness of cigarette smoking for serious psychological distress, stratified by gender. Error bars display 95% confidence intervals. Adjusted for age-groups, family size, housing tenure, marital status, education, equivalent household expenditures, employment contract, and chronic medical conditions

**Fig. 2:**
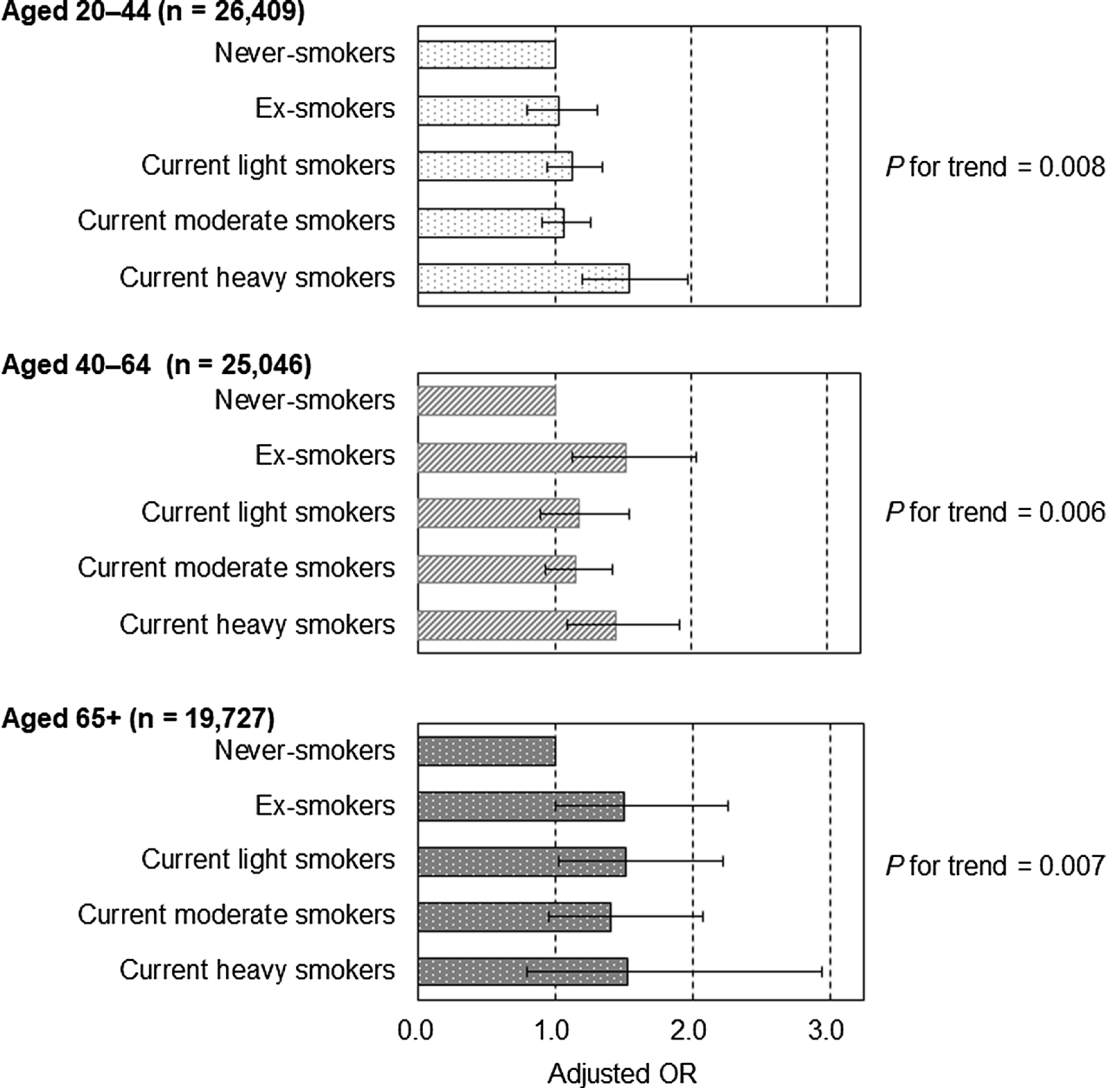
Adjusted odds ratio (OR) of heaviness of cigarette smoking for serious psychological distress, stratified by age-groups. Error bars display 95% confidence intervals. Adjusted for age (5-year increase), family size, housing tenure, marital status, education, equivalent household expenditures, employment contract, and chronic medical conditions

The results of stratified analyses by gender showed that an association between more cigarette smoking and more frequent SPD was significant only in women (*P* for trend < 0.001) but not in men (*P* for trend = 0.649), the significant interaction being that female gender strengthens the association between heaviness of cigarette smoking and SPD (interaction effect of gender × heaviness of cigarette smoking: *P* < 0.001) (Fig. 1). Among women, aORs (95% CIs) of ex-smokers, current light smokers 1–10 CPD, current moderate smokers 11–20 CPD, and current heavy smokers ≥ 21 CPD were 1.22 (0.92–1.63), 1.52 (1.25–1.84), 1.75 (1.46–2.09), and 2.22 (1.59–3.10), respectively, compared to never-smokers. Among men, only current heavy smokers ≥ 21 CPD had a significantly higher aOR for SPD than never-smokers: aOR (95% CI) was 1.32 (1.07–1.64) (Fig. 1). Regarding stratified analyses by age-group, a significant association between heaviness of cigarette smoking and SPD was observed among all age-groups (*P* for trend was 0.008 in ages 20–44, 0.006 in ages 45–64, and 0.007 in ages ≥ 65), with no interaction between heaviness of cigarette smoking and age-group (interaction effect of age-groups × heaviness of cigarette smoking: *P* = 0.431) (Fig. 2).

For stratified analyses by gender and age, women had a significant association between heavier cigarette smoking and higher aOR for SPD, regardless of age (*P* for trend < 0.001 in all age-groups). All current smokers aged 20–44 years (aOR, 1.47 [95% CI, 1.15–1.88] in light smokers; 1.50 [1.16–1.93] in moderate smokers; and 1.88 [1.17–3.02] in heavy smokers), ex-smokers and current smokers with ≥ 11 CPD who were aged 45–64 years (aOR, 1.75 [95% CI, 1.06–2.88] in ex-smokers; 1.64 [1.21–2.22] in moderate smokers; and 2.03 [1.21–3.39] in heavy smokers), and current moderate smokers aged 65 or older (aOR, 2.54 [95% CI, 1.41–4.57]) had a significant higher aOR for SPD than never-smokers, even after controlling for sociodemographic confounders. Among men, a significant association between more cigarette smoking and more common SPD was found in none of the age-groups. Only current heavy smokers aged 20–44 years had a significant higher aOR for SPD (aOR, 1.37 [95% CI, 1.02–1.85]), compared to never-smokers (Table 2).

**Table 2:** Adjusted odds ratios for serious psychological distress according to heaviness of cigarette smoking, stratified by gender and age

Heaviness of cigarette smoking	Young: aged 20–44 years			Middle: aged 45–64 years			Old: aged ≥ 65 years		
	*n*	SPD %	OR^a^ (95% CI)	*n*	SPD %	OR^a^ (95% CI)	*n*	SPD %	OR^a^ (95% CI)
Men (*n* = 33,925)									
Never-smokers	6795	5.3	1.00	6355	2.8	1.00	6404	2.0	1.00
Ex-smokers	907	4.9	1.05 (0.75–1.46)	1080	3.4	1.26 (0.87–1.82)	781	2.7	1.39 (0.87–2.23)
Current light smokers	1573	4.3	0.85 (0.65–1.11)	941	3.1	0.98 (0.65–1.46)	615	3.1	1.40 (0.85–2.30)
Current moderate smokers	2720	4.4	0.85 (0.69–1.07)	2517	2.7	0.86 (0.64–1.15)	841	2.1	0.94 (0.56–1.58)
Current heavy smokers	856	7.0	1.37 (1.02–1.85)^*^	1233	3.7	1.22 (0.87–1.72)	307	2.6	1.25 (0.60–2.62)
			*P* for trend = 0.933			*P* for trend = 0.978			*P* for trend = 0.482
Women (*n* = 37,257)									
Never-smokers	11,051	5.7	1.00	11,087	3.5	1.00	10,208	2.8	1.00
Ex-smokers	531	5.3	0.89 (0.60–1.32)	258	7.0	1.75 (1.06–2.88)^*^	110	5.5	1.80 (0.78–4.18)
Current light smokers	925	9.1	1.47 (1.15–1.88)^*^	610	5.6	1.35 (0.93–1.95)	247	4.5	1.53 (0.82–2.85)
Current moderate smokers	886	9.6	1.50 (1.16–1.93)^*^	777	7.1	1.64 (1.21–2.22)^*^	185	7.0	2.54 (1.41–4.57)^*^
Current heavy smokers	165	13.3	1.88 (1.17–3.02)^*^	188	9.6	2.03 (1.21–3.39)^*^	29	6.9	2.68 (0.63–11.47)
			*P* for trend < 0.001			*P* for trend < 0.001			*P* for trend < 0.001

Light means 1–10 cigarettes per day. Moderate means 11–20 cigarettes per day. Heavy means ≥ 21 cigarettes per day

*CI* Confidence interval, *OR* Odds ratio, *SPD* Serious psychological distress

^*^P < 0.05

^a^Adjusted for age (5-years increase), family size, housing tenure, marital status, education, equivalent household expenditures, employment contract, and chronic medical conditions

For sensitivity analyses which were limited to participants who did not receive treatment for mental disorders, similar results were observed (Additional file 1: Table 4**)**. For the interaction effects of heaviness of cigarette smoking and socio-demographic variables on SPD, the interaction between EHE and heaviness of cigarette smoking was significant only in women (Additional file 1: Table 5). We therefore conducted additional analyses stratified by EHE among women. An association between heavier cigarette smoking and higher aOR for SPD (i.e., *P* for trend) was significant in all EHE groups, but the association between current heavy smokers and SPD was not significant in the middle EHE group (Additional file 1: Table 6). These results suggest that sociodemographic variables have little modifying effect on the association between heaviness of cigarette smoking and SPD.

## Discussion

This study examined gender and age differences in the association between heaviness of cigarette smoking and SPD, using a nationally representative sample of the Japanese population. First, a significant association between heavier cigarette smoking and more prevalent SPD was observed only in women, but not in men. Second, there were no age differences in the association between heaviness of cigarette smoking and SPD. To the best of our knowledge, this is the first study to demonstrate gender differences in the association between heaviness of cigarette smoking and SPD, namely that an association between heavier cigarette smoking and more frequent SPD is observed only in women, regardless of age. Although the prevalence of cigarette smoking is lower among women, our findings suggest that the association between smoking intensity and mental health may be stronger for women than for men.

Previous studies have suggested that an association between heaviness of cigarette smoking and SPD may be significant, but gender differences in the association are unclear. Two Australian studies used a nationally representative sample and reported a positive association between heavier smoking and higher prevalence of SPD [6, 27]. One study found a strong association between smoking and poor mental health including SPD, but limited study participants to young women aged 18–23 years [27]. The other study reported that smoking more CPD was associated with higher levels of psychological distress among persons aged 20 years or more, but performed stratified analyses by neither age nor gender [6]. Regarding a cross-sectional association between presence or absence of current smoking and SPD, several population-based studies [12, 28] showed a significant association only for women, suggesting that smoking is strongly associated with SPD among women, in contrast to men.

We have two potential mechanisms for the association between heaviness of cigarette smoking and SPD in women (i.e., biological and social mechanisms). First, for biological mechanisms, previous studies reported gender differences in the relationship between cortisol reactivity to stress, severity of the hypothalamic–pituitary–adrenal axis dysregulation, and smoking outcomes [29, 30]. These studies suggest that women may be more sensitive to dysfunction of the central nervous system associated with smoking as compared to men [31]. Moreover, a previous study [32] showed that smoking abstinence for one night was strongly associated with greater withdrawal symptoms and greater urges to smoke among female smokers than among male smokers, suggesting that women tend to have more trouble quitting smoking than men [33]. Second, women have a higher risk of mental disorders than men, and one of the risk factors leading to gender differences in mental health may be the gender gap in politics, wages, and unpaid domestic work [34, 35]. According to the World Economic Forum’s Global Gender Gap Index, which is an index measuring the gender-based gap in health, education, economy, and politics, Japan ranks 121st out of 153 countries; in terms of political empowerment, Japan is 144th [35]. According to the latest OECD data, the average gender wage gap in OECD countries is 12.9%, but when the gender wage gap is considered by country, Japan is 23.5%, the second highest among OECD member countries [36]. Furthermore, in Japan, women spend more than four times as much time as men on unpaid domestic work; in Norway and the United States, the amount of time that women spend is about twice that of men [35]. These international comparative statistics suggest that Japan’s gender gap is at the largest level internationally, which might contribute to deterioration in the mental health of Japanese women. Third, the prevalence of smoking in Japan is characterized by a great gender difference, being higher in men and lower in women compared to the average smoking prevalence by gender in Western countries [37]. Several researchers have pointed out that the Japanese have a tendency to be tolerant toward male smoking but disapproving of female smoking [38, 39], and that Japan has a shame culture based on collectivism [40]. Shame is a reaction to the criticism of others [40], and is associated with adverse effects on mental health including SPD [41, 42]. Since social expectations in Japan are that women should not smoke, Japanese female smokers are more likely to experience social disapproval, which may in turn induce feelings of shame. In Japan, therefore, the mental health of female smokers may tend to be more affected than that of male smokers, possibly contributing to a stronger association between heaviness of cigarette smoking and SPD in women than in men.

Our study did not find significant differences in the association between heaviness of cigarette smoking and SPD for age-groups. Two previous studies conducted stratified analyses by gender and age, but had inconsistent results; the association between current smoking and SPD was significant for people aged 40–64 years, but not for those aged 65 years or more, regardless of gender [5], or was significant for men aged ≥ 50 years and for women aged ≥ 20 years [3]. The age modifying effect on the association between smoking and SPD appears inconclusive.

The strengths of this study include a high response rate (79.6%), a representative sample of the Japanese people, a large sample size, and an adequate adjustment for sociodemographic confounders. On the other hand, this study has several limitations. First, we cannot investigate causality because it was a cross-sectional study. Second, our findings were based on self-assessment of heaviness of smoking and SPD, and therefore were exposed to self-reporting biases. Our findings should have been checked against objective data such as measures of nicotine dependence. Third, although our study included sufficient covariates based on previous studies, there is the possibility of unmeasured confounding and residual confounding. A recent study reveals that an association between psychological distress and current smoking is modified by socioeconomic stressors such as food insecurity [24]. Moreover, the observed association between heaviness of smoking and SPD may be due to shared genetic factors such as the CHRNA5-A3-B4 gene cluster, which is reported to be associated with heaviness of cigarette smoking [43]. To address bias from unobserved confounders, future research should use instrumental variables including a Mendelian randomization analysis. Fourth, although the 2013 data used in this study are the latest available data, as of December 2020, it is seven years old. According to the summary of results of raw data of the CSLC published by the MHLW [44], the adult smoking prevalence in men and women was reported to be 33.7% and 10.7% in 2013, 31.1% and 9.5% in 2016, and 28.8% and 8.8% in 2019, respectively. We should note that the aggregated results based on anonymized data do not completely match the aggregated results based on raw data. Because adult smoking prevalence in Japan is decreasing year by year, future studies need to re-analyze the association between heaviness of cigarette smoking and SPD with the latest data.

Regarding implications of this study, our findings suggest that female smokers with SPD may have a greater need of support for smoking cessation as well as access to mental health treatment. Clinicians should be made aware that the association between heaviness of cigarette smoking and mental health may be stronger for women than for men. Policymakers should highlight that effective tobacco control efforts should include a focus on women with mental health problems.

## Conclusions

This nationally representative cross-sectional study in Japan demonstrated that an association between greater heaviness of cigarette smoking and higher prevalence of SPD was significant only among women, but not among men. In addition, the association between heaviness of cigarette smoking and SPD was not affected by age. Our findings underline the importance of considering gender when attempting to reduce adult smoking prevalence for mental health maintenance in Japan.

## Supplementary Information

**Additional file 1.** **Supplementary Table 1**. Characteristics of the 33,925 male subjects. **Supplementary Table 2**. Characteristics of the 37,257 female subjects. **Supplementary Table 3**. Prevalence of serious psychological distress according to age, gender, and basic characteristics. **Supplementary Table 4**. Sensitivity analyses limited to participants without medical treatment for mental disorders; adjusted odds ratios for serious psychological distress, stratified by gender and age. **Supplementary Table 5**. Modifying effect of socio-demographic variables on the relationship between heaviness of cigarette smoking and serious psychological distress. **Supplementary Table 6**. Adjusted odds ratios of heaviness of cigarette smoking for serious psychological distress in stratified analyses by equivalent household expenditures among women.

## Data Availability

Data are available from the Ministry of Health, Labour and Welfare, Japan (https://www.mhlw.go.jp/toukei/itaku/tokumei.html) for researchers who obtain approval to use the anonymous data in accordance with Article 36 of the Statistics Law of Japan.

## Abbreviations

aOR: Adjusted odds ratio
CI: Confidence interval
CPS: Cigarettes per day
CSLC: Comprehensive Survey of Living Conditions
EHE: Equivalent household expenditures
K6: Kessler 6-item Psychological Distress Scale
MHLW: Ministry of Health, Labour and Welfare
SPD: Serious psychological distress
VIF: Variance inflation factor
