# Supplementary material for: Association between heaviness of cigarette smoking and serious psychological distress is stronger in women than in men: a nationally representative cross-sectional survey in Japan
Source: Harm Reduct J. 2021 Mar 4;18:27. doi: 10.1186/s12954-021-00469-5 (PMC7931554; doi:10.1186/s12954-021-00469-5)
Supplement: Supplementary file 1 — Additional file 1. Supplementary Table 1. Characteristics of the 33,925 male subjects. Supplementary Table 2. Characteristics of the 37,257 female subjects. Supplementary Table 3. Prevalence of serious psychological distress according to age, gender, and basic characteristics. Supplementary Table 4. Sensitivity analyses limited to participants without medical treatment for mental disorders; adjusted odds ratios for serious psychological distress, stratified by gender and age. Supplementary Table 5. Modifying effect of socio-demographic variables on the relationship between heaviness of cigarette smoking and serious psychological distress. Supplementary Table 6. Adjusted odds ratios of heaviness of cigarette smoking for serious psychological distress in stratified analyses by equivalent household expenditures among women. [file 12954_2021_469_MOESM1_ESM.docx]

**Supplementary Table 1** Characteristics of the 33,925 male subjects

|  |  | 20–44 years | 45–64 years | ≥65 years |
| --- | --- | --- | --- | --- |
|  |  | (n=12,851) | (n=12,126) | (n=8,948) |
|  |  | n (%) | n (%) | n (%) |
| Family size | |  |  |  |
|  | 1: living alone | 1,691 (13.2) | 1,482 (12.2) | 977 (10.9) |
|  | 2 | 1,835 (14.3) | 3,123 (25.8) | 4,572 (51.1) |
|  | 3–4 | 7,202 (56.0) | 5,901 (48.7) | 2,614 (29.2) |
|  | ≥5 | 2,123 (16.5) | 1,620 (13.4) | 785 (8.8) |
| Housing tenure | |  |  |  |
|  | Owner-occupiers | 7,665 (59.6) | 9,392 (77.5) | 7,614 (85.1) |
|  | Renters | 5,186 (40.4) | 2,734 (22.5) | 1,334 (14.9) |
| Marital status | |  |  |  |
|  | Married | 6,474 (50.4) | 9,559 (78.8) | 7,592 (84.8) |
|  | Never-married | 6,106 (47.5) | 1,679 (13.8) | 264 (3.0) |
|  | Widowed/divorced | 271 (2.1) | 888 (7.3) | 1,092 (12.2) |
| Education (years of schooling) | |  |  |  |
|  | Junior high school (≤9 years) | 636 (4.9) | 1,004 (8.3) | 2,523 (28.2) |
|  | High school (10–12 years) | 4,211 (32.8) | 4,968 (41.0) | 3,371 (37.7) |
|  | Junior college (13–15 years) | 2,019 (15.7) | 1,148 (9.5) | 342 (3.8) |
|  | College or higher (≥16 years) | 4,851 (37.7) | 3,783 (31.2) | 1,551 (17.3) |
|  | Missing | 1,134 (8.8) | 1,223 (10.1) | 1,161 (13.0) |
| Equivalent household expenditures (Japanese thousand yen per month) | | | |  |
|  | Low: lower tertile (≤105) | 4,593 (35.7) | 3,408 (28.1) | 2,911 (32.5) |
|  | Middle: middle tertile (106–156) | 4,205 (32.7) | 3,675 (30.3) | 2,625 (29.3) |
|  | High: upper tertile (≥157) | 3,537 (27.5) | 4,523 (37.3) | 3,045 (34.0) |
|  | Missing | 516 (4.0) | 520 (4.3) | 367 (4.1) |
| Employment contract | |  |  |  |
|  | Regular employees | 8,418 (65.5) | 6,353 (52.4) | 363 (4.1) |
|  | Part-time employees | 796 (6.2) | 537 (4.4) | 459 (5.1) |
|  | Temporary/contract employees | 603 (4.7) | 991 (8.2) | 413 (4.6) |
|  | Self-employed | 1,398 (10.9) | 2,607 (21.5) | 1,776 (19.8) |
|  | Non-working | 1,377 (10.7) | 1,423 (11.7) | 5,470 (61.1) |
|  | Missing | 259 (2.0) | 215 (1.8) | 467 (5.2) |
| Chronic medical conditions under treatment | | |  |  |
|  | Absent | 12,512 (97.4) | 9,410 (77.6) | 4,830 (54.0) |
|  | Present | 302 (2.4) | 2,682 (22.1) | 4,069 (45.5) |
|  | Missing | 37 (0.3) | 34 (0.3) | 49 (0.5) |

**Supplementary Table 2** Characteristics of the 37,257 female subjects

|  |  | 20–44 years | 45–64 years | ≥65 years |
| --- | --- | --- | --- | --- |
|  |  | (n=13,558) | (n=12,920) | (n=10,779) |
|  |  | n (%) | n (%) | n (%) |
| Family size | |  |  |  |
|  | 1: living alone | 963 (7.1) | 1,038 (8.0) | 2,302 (21.4) |
|  | 2 | 2,061 (15.2) | 4,103 (31.8) | 4,647 (43.1) |
|  | 3–4 | 7,937 (58.5) | 6,248 (48.4) | 2,765 (25.7) |
|  | ≥5 | 2,597 (19.2) | 1,531 (11.8) | 1,065 (9.9) |
| Housing tenure | |  |  |  |
|  | Owner-occupiers | 8,339 (61.5) | 10,264 (79.4) | 8,974 (83.3) |
|  | Renters | 5,219 (38.5) | 2,656 (20.6) | 1,805 (16.7) |
| Marital status | |  |  |  |
|  | Married | 7,573 (55.9) | 10,138 (78.5) | 6,030 (55.9) |
|  | Never-married | 5,193 (38.3) | 944 (7.3) | 351 (3.3) |
|  | Widowed/divorced | 792 (5.8) | 1,838 (14.2) | 4,398 (40.8) |
| Education (years of schooling) | |  |  |  |
|  | Junior high school (≤9 years) | 436 (3.2) | 909 (7.0) | 3,754 (34.8) |
|  | High school (10–12 years) | 4,115 (30.4) | 5,947 (46.0) | 4,378 (40.6) |
|  | Junior college (13–15 years) | 4,502 (33.2) | 3,346 (25.9) | 903 (8.4) |
|  | College or higher (≥16 years) | 3,316 (24.5) | 1,318 (10.2) | 365 (3.4) |
|  | Missing | 1,189 (8.8) | 1,400 (10.8) | 1,379 (12.8) |
| Equivalent household expenditures (Japanese thousand yen per month) | | | |  |
|  | Low: lower tertile (≤105) | 4,843 (35.7) | 3,552 (27.5) | 3,967 (36.8) |
|  | Middle: middle tertile (106–156) | 4,546 (33.5) | 3,914 (30.3) | 3,149 (29.2) |
|  | High: upper tertile (≥157) | 3,595 (26.5) | 4,884 (37.8) | 3,220 (29.9) |
|  | Missing | 574 (4.2) | 570 (4.4) | 443 (4.1) |
| Employment contract | |  |  |  |
|  | Regular employees | 4,276 (31.5) | 2,256 (17.5) | 135 (1.3) |
|  | Part-time employees | 3,168 (23.4) | 3,604 (27.9) | 469 (4.4) |
|  | Temporary/contract employees | 996 (7.3) | 768 (5.9) | 91 (0.8) |
|  | Self-employed | 886 (6.5) | 1,610 (12.5) | 1,132 (10.5) |
|  | Non-working | 4,048 (29.9) | 4,522 (35.0) | 8,586 (79.7) |
|  | Missing | 184 (1.4) | 160 (1.2) | 366 (3.4) |
| Chronic medical conditions under treatment | | |  |  |
|  | Absent | 13,316 (98.2) | 10,874 (84.2) | 6,470 (60.0) |
|  | Present | 199 (1.5) | 1,993 (15.4) | 4,259 (39.5) |
|  | Missing | 43 (0.3) | 53 (0.4) | 50 (0.5) |

**Supplementary Table 3** Prevalence of serious psychological distress according to age, gender, and basic characteristics

|  |  | Men (n=33,925) | | |  | Women (n=37,257) | | |
| --- | --- | --- | --- | --- | --- | --- | --- | --- |
|  | | 20–44 years | 45–64 years | ≥65 years |  | 20–44 years | 45–64 years | ≥65 years |
| Family size | |  |  |  |  |  |  |  |
|  | 1: living alone | 8.8% | 4.9% | 3.9% |  | 11.2% | 6.0% | 3.3% |
|  | 2 | 5.5% | 3.0% | 1.9% |  | 6.6% | 3.5% | 2.8% |
|  | 3–4 | 4.5% | 2.4% | 2.0% |  | 5.9% | 4.0% | 3.3% |
|  | ≥5 | 3.9% | 3.0% | 2.2% |  | 5.1% | 3.5% | 2.3% |
| Housing tenure | |  |  |  |  |  |  |  |
|  | Owner-occupiers | 4.2% | 2.5% | 1.9% |  | 6.0% | 3.3% | 2.7% |
|  | Renters | 6.4% | 4.5% | 3.7% |  | 6.7% | 6.4% | 4.4% |
| Marital status | |  |  |  |  |  |  |  |
|  | Married | 3.7% | 2.4% | 1.9% |  | 4.8% | 3.3% | 2.4% |
|  | Never-married | 6.5% | 4.7% | 2.3% |  | 7.9% | 6.4% | 3.1% |
|  | Widowed/divorced | 6.3% | 5.3% | 4.2% |  | 9.6% | 6.0% | 3.7% |
| Education (years of schooling) | | | | |  |  |  |  |
|  | ≤9 years | 8.5% | 3.4% | 2.9% |  | 13.1% | 6.4% | 3.6% |
|  | 10–12 years | 5.2% | 3.0% | 1.8% |  | 7.0% | 3.7% | 2.4% |
|  | 13–15 years | 5.3% | 3.5% | 2.9% |  | 5.2% | 3.4% | 2.4% |
|  | ≥16 years | 4.4% | 2.3% | 1.2% |  | 5.6% | 3.9% | 1.9% |
|  | Missing | 5.5% | 3.8% | 2.6% |  | 7.1% | 4.6% | 3.6% |
| Equivalent household expenditures (Japanese thousand yen per month) | | | | | | | | |
|  | Low (≤105) | 4.9% | 3.2% | 2.7% |  | 6.1% | 5.0% | 3.0% |
|  | Middle (106–156) | 5.2% | 3.2% | 2.3% |  | 6.2% | 4.0% | 2.8% |
|  | High (≥157) | 5.0% | 2.5% | 1.3% |  | 6.1% | 3.0% | 2.8% |
|  | Missing | 5.7% | 3.2% | 3.3% |  | 8.4% | 5.2% | 4.5% |
| Employment contract | |  |  |  |  |  |  |  |
|  | Permanent | 4.1% | 2.5% | 1.1% |  | 5.6% | 2.9% | 2.2% |
|  | Part-time | 6.5% | 2.4% | 0.9% |  | 6.0% | 3.3% | 1.3% |
|  | Temporary/contract | 7.3% | 2.2% | 1.9% |  | 6.3% | 2.6% | 4.4% |
|  | Self-employed | 3.6% | 2.1% | 1.4% |  | 4.5% | 3.9% | 2.0% |
|  | Non-working | 10.6% | 7.0% | 2.5% |  | 7.3% | 5.1% | 3.1% |
|  | Missing | 5.4% | 2.8% | 3.6% |  | 9.2% | 6.9% | 3.6% |
| Chronic medical conditions under treatment | | | | |  |  |  |  |
|  | Absent | 4.9% | 2.8% | 2.2% |  | 6.1% | 3.7% | 2.6% |
|  | Present | 11.3% | 3.5% | 2.2% |  | 12.6% | 5.7% | 3.4% |
|  | Missing | 5.4% | 8.8% | 0.0% |  | 7.0% | 0.0% | 6.0% |

**Supplementary Table 4** Sensitivity analyses limited to participants without medical treatment for mental disorders; adjusted odds ratios for serious psychological distress, stratiﬁed by gender and age

| Heaviness of  cigarette smoking | | Young: aged 20–44 years | | | Middle: aged 45–64 years | | | Old: aged ≥65 years | | |
| --- | --- | --- | --- | --- | --- | --- | --- | --- | --- | --- |
|  |  | n | SPD % | OR^a^ (95% CI) | n | SPD % | OR^a^ (95% CI) | n | SPD % | OR^a^ (95% CI) |
| Men (n=33,273) | |  |  |  |  |  |  |  |  |  |
|  | Never-smokers | 6,618 | 4.5 | 1.00 | 6,239 | 2.4 | 1.00 | 6,317 | 1.9 | 1.00 |
|  | Ex-smokers | 895 | 4.6 | 1.17 (0.83–1.64) | 1,060 | 3.0 | 1.27 (0.86–1.88) | 775 | 2.3 | 1.23 (0.74–2.03) |
|  | Current light smokers | 1,556 | 4.0 | 0.92 (0.69–1.22) | 910 | 2.6 | 0.98 (0.63–1.53) | 609 | 3.0 | 1.37 (0.83–2.28) |
|  | Current moderate smokers | 2,659 | 3.6 | 0.84 (0.66–1.07) | 2,464 | 2.3 | 0.85 (0.62–1.16) | 823 | 1.9 | 0.88 (0.51–1.51) |
|  | Current heavy smokers | 830 | 6.4 | 1.51 (1.10–2.07)^*^ | 1,212 | 3.2 | 1.22 (0.85–1.76) | 306 | 2.6 | 1.29 (0.61–2.70) |
|  |  |  |  | *P* for trend=0.802 |  |  | *P* for trend=0.986 |  |  | *P* for trend=0.607 |
| Women (n=36,292) | |  |  |  |  |  |  |  |  |  |
|  | Never-smokers | 10,771 | 4.9 | 1.00 | 10,820 | 3.0 | 1.00 | 10,015 | 2.5 | 1.00 |
|  | Ex-smokers | 513 | 4.3 | 0.83 (0.54–1.29) | 247 | 6.9 | 2.01 (1.20–3.36)^*^ | 104 | 4.8 | 1.72 (0.69–4.30) |
|  | Current light smokers | 886 | 7.4 | 1.34 (1.02–1.76)^*^ | 585 | 4.3 | 1.18 (0.77–1.81) | 240 | 4.6 | 1.80 (0.96–3.37) |
|  | Current moderate smokers | 836 | 6.9 | 1.23 (0.92–1.66) | 739 | 5.7 | 1.51 (1.07–2.12)^*^ | 178 | 5.6 | 2.18 (1.12–4.23)^*^ |
|  | Current heavy smokers | 155 | 12.3 | 2.01 (1.21–3.34)^*^ | 174 | 8.0 | 1.99 (1.12–3.53)^*^ | 29 | 6.9 | 2.91 (0.68–12.49) |
|  |  |  |  | *P* for trend=0.004 |  |  | *P* for trend=0.001 |  |  | *P* for trend=0.001 |

Light means 1–10 cigarettes per day. Moderate means 11–20 cigarettes per day. Heavy means ≥21 cigarettes per day

^a^Adjusted for age (5–years increase), family size, housing tenure, marital status, education, equivalent household expenditures, employment contract, and chronic medical conditions

*CI* Conﬁdence interval, *OR* Odds ratio, *SPD* Serious psychological distress

^*^*P*<0.05

**Supplementary Table 5** Modifying effect of socio–demographic variables on the relationship between heaviness of cigarette smoking and serious psychological distress

|  |  | *P* for interaction | |
| --- | --- | --- | --- |
|  |  | Men | Women |
| Interaction effect of heaviness of cigarette smoking | |  |  |
|  | × family size | 0.277 | 0.297 |
|  | × housing tenure | 0.584 | 0.150 |
|  | × marital status | 0.097 | 0.093 |
|  | × education | 0.168 | 0.661 |
|  | × equivalent household expenditures | 0.558 | 0.003 |
|  | × employment contract | 0.599 | 0.833 |
|  | × chronic medical conditions | 0.172 | 0.627 |

The model was adjusted for age groups, family size, housing tenure, marital status, education, equivalent household expenditures, employment contract, and chronic medical conditions.

**Supplementary Table 6** Adjusted odds ratios of heaviness of cigarette smoking for serious psychological distress in stratiﬁed analyses by equivalent household expenditures among women

| EHE | Heaviness of cigarette smoking | n | SPD % | OR^a^ (95% CI) |
| --- | --- | --- | --- | --- |
| Low | Never-smokers | 10,485 | 4.2 | 1.00 |
|  | Ex-smokers | 312 | 5.8 | 1.21 (0.74–1.98) |
|  | Current light smokers 1–10 CPD | 675 | 8.0 | 1.64 (1.21–2.22)^*^ |
|  | Current moderate smokers 11–20 CPD | 705 | 7.2 | 1.47 (1.07–2.00)^*^ |
|  | Current heavy smokers 21+ CPD | 125 | 20.8 | 4.86 (3.08–7.66)^*^ |
|  |  |  |  | *P* for trend <0.001 |
| Middle | Never-smokers | 10,010 | 4.1 | 1.00 |
|  | Ex-smokers | 283 | 7.8 | 1.61 (1.02–2.53)^*^ |
|  | Current light smokers 1–10 CPD | 572 | 6.3 | 1.25 (0.87–1.80) |
|  | Current moderate smokers 11–20 CPD | 586 | 8.2 | 1.67 (1.21–2.31)^*^ |
|  | Current heavy smokers 21+ CPD | 115 | 3.5 | 0.62 (0.23–1.71) |
|  |  |  |  | *P* for trend=0.013 |
| High | Never-smokers | 10,380 | 3.5 | 1.00 |
|  | Ex-smokers | 270 | 3.7 | 0.90 (0.47–1.71) |
|  | Current light smokers 1–10 CPD | 437 | 5.9 | 1.46 (0.96–2.23) |
|  | Current moderate smokers 11–20 CPD | 450 | 9.8 | 2.70 (1.92–3.80)^*^ |
|  | Current heavy smokers 21+ CPD | 107 | 10.3 | 2.60 (1.36–4.97)^*^ |
|  |  |  |  | *P* for trend <0.001 |

^a^Adjusted for age (5–years increase), family size, housing tenure, marital status, education, employment contract, and chronic medical conditions

*CI* Conﬁdence interval, *CPD* Cigarettes per day, *EHE* Equivalent household expenditures, *OR* Odds ratio, *SPD* Serious psychological distress

^*^*P*<0.05
